# Ultraviolet Photodetector Based on Poly(3,4-Ethylenedioxyselenophene)/ZnO Core–Shell Nanorods p-n Heterojunction

**DOI:** 10.1186/s11671-022-03705-4

**Published:** 2022-07-25

**Authors:** Aygul Kadir, Ruxangul Jamal, Tursun Abdiryim, Xiong Liu, Hujun Zhang, Nawrzhan Serkjan, Dongna Zou, Ya jun Liu

**Affiliations:** 1 State Key Laboratory of Chemistry and Utilization of Carbon Based Energy Resources, College of Chemistry, Xinjiang University, Urumqi, 830017 Xinjiang People’s Republic of China; 2 State Key Laboratory of Chemistry and Utilization of Carbon Based Energy Resources, Key Laboratory of Petroleum and Gas Fine Chemicals, Ministry of Education, College of Chemical Engineering, Xinjiang University, Urumqi, 830017 Xinjiang People’s Republic of China

**Keywords:** Poly(3,4-ethylenedioxyselenophene), Ultraviolet photodetector, Electrochemical deposition method, p-n heterojunction

## Abstract

In this work, we successfully assembled an organic–inorganic core–shell hybrid p-n heterojunction ultraviolet photodetector by the electropolymerization deposition of poly(3,4-ethylenedioxyselenophene) (PEDOS) on the surface of zinc oxide nanoarrays (ZnO NRs). The structures of composite were confirmed by FTIR, UV–Vis, XRD and XPS. Mott–Schottky analysis was used to study the p-n heterojunction structure. The photodetection properties of ZnO NRs/PEDOS heterojunction ultraviolet photodetector were systematically investigated current–voltage (I–V) and current–time (I–t) analysis under different bias voltages. The results showed that PEDOS films uniformly grew on ZnO NRs surface and core–shell structure was formed. The p-n heterojunction structure was formed with strong built-in electric field between ZnO NRs and PEDOS. Under the irradiation of UV light, the device showed a good rectification behavior. The responsivity, detection rate and the external quantum efficiency of the ultraviolet photodetector reached to 247.7 A/W, 3.41 × 10^12^ Jones and 84,000% at 2 V bias, respectively. The rise time (*τ*_r_) and fall time (*τ*_f_) of ZnO NRs/PEDOS UV photodetector were obviously shortened compared to ZnO UV photodetector. The results show that the introduction of PEDOS effectively improves the performance of the UV photodetector.

**Supplementary Information:**

The online version contains supplementary material available at 10.1186/s11671-022-03705-4.

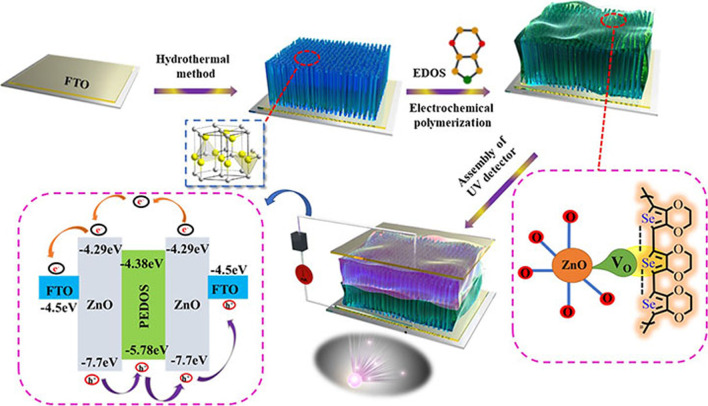

## Introduction

Recently, ultraviolet photodetectors (UV PDs) has been widely used in the following fields, including ultraviolet guidance, fire detection, optical communications and medical applications [1–4]. Among the wide-bandgap semiconductors materials, ZnO is one of the best candidate materials for UV PDs due to its unique performance, such as wide direct bandgap (~ 3.3 eV) and high exciton binding energy (60 meV) [5, 6], with excellent photoelectric conversion characteristics, thermal stability, nontoxic, easy preparation and inexpensive [1, 7, 8]. However, ZnO-based UV PDs still have the problems that weak light response [9] and response time longer [10], which limits its application in UV PDs [11]. Therefore, reducing the carrier composite rate and extending the carrier life are important for improving the ZnO-based UV PDs [12, 13]. Recent studies have shown that nanostructured ZnO is beneficial for photogenic carrier transport, nanocomposite materials based on p-n heterojunction contribute to the improvement of photoconductivity [14, 15], and the construction of nanostructured ZnO p-n heterojunction is expected to be the key to solve the problem in these UV PD issues. However, the quality of the p-n heterojunctions in UV PDs really determines the device performance [16]. Therefore, it is important to construct a suitable p-n heterojunction to improve the performance of UV PD [17].

To obtain high-quality ZnO-based p-n heterojunction UV PDs, it is also important to select appropriate p-type materials [12]. Recently, conductive polymers have been received extensive attention for applications in UV PDs, batteries, supercapacitors and electrochemical detection due to their exciting material properties such as excellent hole transport capacity, high transmittance in the UV–visible region, easy processing, excellent flexibility, conjugated π-electrons, good stability solution-processed deposition technique and low material cost. Therefore, conjugated polymers are shown as a potential candidate materials after combining p-n heterojunction photovoltaic devices with n-type semiconductors [18].

Common conductive polymers polyaniline (PANI) [10], P3HT [19] and poly(3,4-ethylenedioxythiophene) (PEDOT) [20] and other organic materials have been widely applied in the UV PDs, which as typical p-type materials. Chen et al. [17] successfully prepared polyaniline/zinc oxide heterojunction UV PD that using the PANI and ZnO as p-type and n-type materials, respectively. Wang et al. [15] fabricated a UV detector composed of ZnO nanorods/polyaniline (PANI) nanofibers p-n type heterojunctions. It was found that the recombination of p-type polymer and n-type ZnO would be favorable to the directional migration of photogenerated electrons and holes. Yu et al. [21] designed different conductive polymers/inorganic hybrid materials by simple oxidative polymerization method and compared their properties. The PANI/Se composite showed higher photoelectric characteristics (switching performance 1.1 × 10^3^ and response rate reached 120 mA/W). This implies that the selection of p-type polymer has an obvious effect on the UV photodetector performance of p-n heterojunction [22].

Among the conductive polymers, poly(3,4-ethylenedioxyselenophene) (PEDOS) has some outstanding properties such as low band gap, lower aromaticity, abundant quinoid structure on polymer chain, strong intermolecular Se···Se interactions and rigidity [23]. Therefore, PEDOS for photovoltaics and organic field effect transistors application has been achieved great consideration. PEDOS is a π-conjugated conducting polymer and exhibited a good hole-transport ability. Furthermore, PEDOS shows a narrower band gap (1.4 eV) and redox potential compared to the PEDOT (1.6 eV); because the Se atoms have larger radius and easy to polarized than S atoms, it is allowed the selenophene rings to anchor more charges than the thiophene rings [24]. However, there are very few reports on the application of PEDOS as p-type conducting polymers in UV photodetectors.

In this paper, a novel ZnO NRs/PEDOS p-n heterojunction has been successfully designed and prepared. The combination of n-type ZnO NRs with p-type PEDOS is expect to form p-n heterojunction and the built-in field by interaction between Se atoms in PEDOS with oxygen vacancies of ZnO NRs. In addition, p-n heterojunction between PEDOS and ZnO NRs will realize the efficiently separation of e–h+ pairs which favorable to achieving high-performance UV PD. Compared with PEDOT, the low energy band gap and redox potential of PEDOS can improve the UV photoelectric detection performance of PEDOS modified ZnO NRs. On this basis, the structure, morphology and optical response characteristics of ZnO NRs/PEDOS UV PD are systematically studied to evaluate its optical detection performance.

## Experimental

### Chemicals

Butan-2,3-dione, trimethylorthoformiate, *p*-toluenesulfonic acid (p-TSA), ethylene glycol, methanol, hydroquinone, ammonium dihydrogen phosphate, natrium aceticum and anhydrous magnesium sulfate were obtained from J&K Scientific Ltd. Selenium powder, SO_2_Cl_2_, tetrabutyl hexafluorophosphate (TBAPF_6_), zinc acetate and zinc nitrate were purchased from Aladdin. Nippon Sheet Glass Co. Ltd was provided the FTO glass (2–10 Ω/square of sheet resistance). All other chemicals can be used without any purification.

### Structure Characterization

The FTIR spectrum of the prepared samples was recorded by the Fourier infrared spectrometer (BRUKER-QEUINOX-55). The absorbance of the sample on the FTO was measured through the UV–Vis spectrometer (UV2450). The morphology information of the samples can be observed with the scanning electron microscope (SEM, SO8010, Japan). The chemical states of PEDOS and ZnO NRs/PEDOS were investigated by X-ray photoelectron spectroscopy (XPS, ESCALAB 250Xi). Raman spectrum was recorded with a Bruker Vertex 70 FT Infrared Spectrometer and the range of 300–4000 cm^−1^ with *λ*_ex_ = 785 nm. The transmission electron microscopy (TEM) images are obtained using a FEI Talos F200i.

### Preparation of the ZnO NRs/PEDOS Nanocomposites

The preparation process of ZnO NRs and monomer of EDOS is in the Additional file 1.

ZnO NRs/PEDOS nanocomposite was prepared by electrochemical deposition methods. The FTO glass with ZnO NRs is used as working electrode, and PEDOS film was electrochemical deposition on ZnO NRs by the cyclic voltammetry (CV) method as the solution of dichloromethane containing 0.01 M EDOS and 0.05 M TBAPF_6_ between 0~1.6 V for four cycles (sweeping rate of 100 mV/s). The electrodeposited film was cleaned with solvent of dichloromethane for 3 times. The prepared film was labeled as ZnO NRs/PEDOS.

### Assembly of UV PD

The preparation route of the ZnO NRs/PEDOS device is shown in graphical abstract. (Assembly of the detailed in the Additional file 1.)

## Results and Discussions

**Fig. 1:**
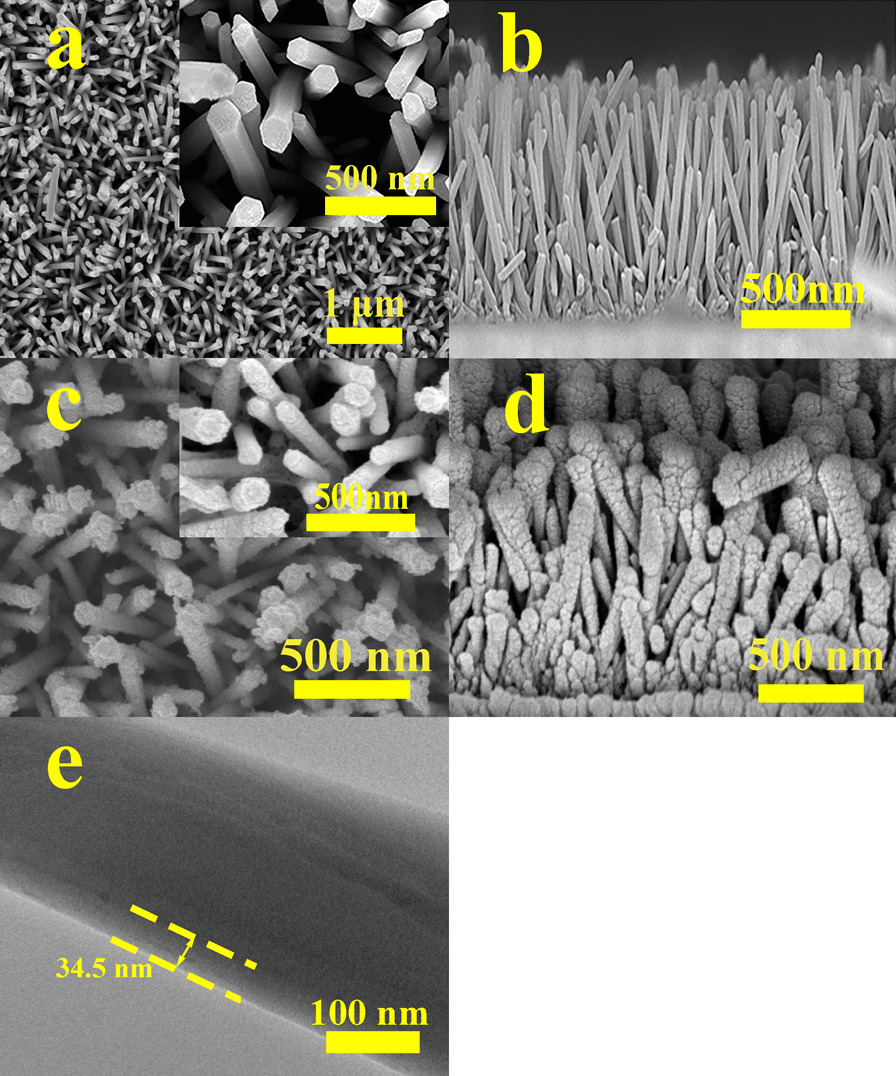
**a** Top-view SEM images of ZnO NRs (inset is a local amplification of the ZnO NRs). **b** Cross-section diagram of ZnO NRs. **c** Top-view SEM images of ZnO NRs/PEDOS (inset is a local amplification of the ZnO NRs/PEDOS). **d** Cross-section diagram of ZnO NRs/PEDOS. **e** TEM image of the ZnO NRs/PEDOS core–shell heterostructure

Figure 1 shows the apparent morphology analysis of ZnO NRs/PEDOS and ZnO NRs by scanning electron microscope. Figure 1a is the top view of the ZnO NRs, which shows smooth surface area and a highly ordered regular hexagonal shape [25, 26]. The inset exhibits a typical hexagonal of ZnO NRs with a diameter of about 86 nm. It is not difficult to find that there is a certain distance between the adjacent ZnO NRs, and it is good for the uniform coating of the PEDOS on the ZnO NRs. Figure 1b clearly shows the cross-section diagram of ZnO NRs vertical growth on the FTO surface.

Figure 1c exhibits that a top view of the ZnO NRs/PEDOS composite and found that the surface of ZnO NRs becomes rough and the diameter of nanorods increases, indicating that PEDOS has successfully grown on ZnO NRs. Figure 1d displays the cross-section diagram of composite, and it can be observed that the PEDOS uniformly covers the ZnO surface to formed a core–shell structure with ZnO NRs, in which the shell is PEDOS, while the core is ZnO NRs.It is known that such core–shell structure assists in reducing the dangling bonds on the surface by passivation of the shell, which can further enhance the device performance [27].

Figure 1e is TEM of the ZnO NRs/PEDOS, and it further proves that the ZnO NRs/PEDOS composite forms a core–shell structure, of which the PEDOS is tightly and densely coated on the ZnO NRs, with the average thickness of the PEDOS of 34.5 nm. This core–shell structure enables ZnO NRs to form a good heterojunction with PEDOS, which is favorable for the generation, transmission and separation of photogenic electron hole pairs.

**Fig. 2:**
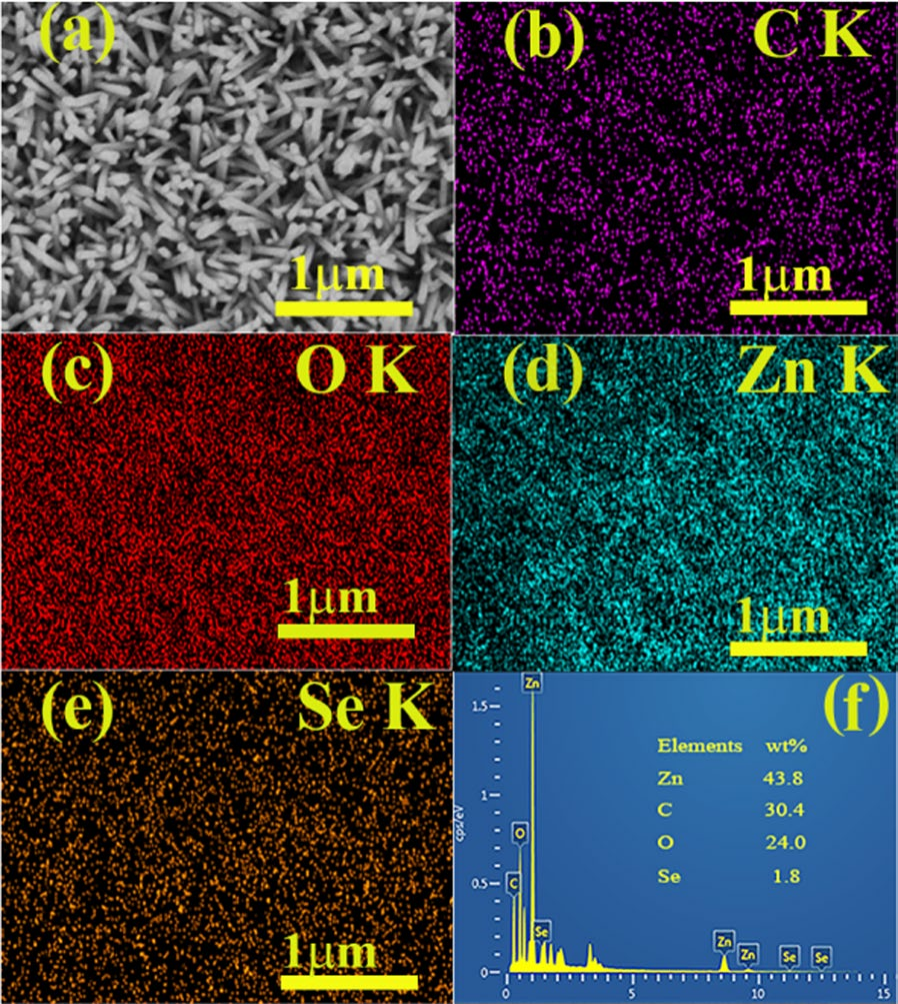
**a** The top view SEM imageof ZnO NRs/PEDOS. **b**–**e** The element mapping images of ZnO NRs/PEDOS: **b** element C, **c** element O, **d** element Zn, **e** element Se. **f** EDS spectrum of ZnO NRs / PEDOS

Figure 2(a)–(e) describes single element mapping of C, O, Se and Zn atoms in the prepared ZnO NRs/PEDOS. The mapping diagram confirms that the elements are evenly distributed. Figure 2(f) shows the EDS of ZnO NRs/PEDOS. It can be found that the peaks of C, Zn, O, and Se are performed, and these results are agreed with the mapping test, which demonstrating the successfully preparation of the composite.

Additional file 1: Figure S3a is the ultraviolet visible (UV–Vis) of the samples, and there is a broad peak at 450–650 nm for the π–π* transitions in the PEDOS main chains. The pure ZnO NRs shows obvious absorption from 350 to 400 nm. For ZnO NRs/PEDOS, a broad peak appears in the range of 500–800 nm [28], which is due to the π–π* transitions of the main chain of PEDOS. The results indicate that the ZnO NRs/PEDOS shows strong UV absorption than that of ZnO NRs, which implies that the conjugation degree of ZnO NRs/PEDOS is enhanced. This can be able to further improve the UV photoresponse of the prepared hybrid heterojunction.

Additional file 1: Fig. S3b displays the infrared spectra of ZnO NRs, PEDOS and ZnO NRs/PEDOS. The broad peak ~ 3400 cm^−1^ is attributed to the characteristic stretching vibration of the O–H bond, which is may be the adsorption of water on the ZnO at ambient [29]. The infrared spectra of ZnO NRs/PEDOS are consistent with that of PEDOS. The characteristic peaks of the C=C asymmetric structure and the C–C tensile vibration in EDOS are located at 1512, 1311 and 1288 cm^−1^. The peaks at ~ 1183 and ~ 1049 cm^−1^ are contributed to the C–O–C flexural characteristic vibration on the ethylenedioxy group, and at 828 and 680 cm^−1^ peaks are the stretching vibration of the C–Se–C on the selenophene ring [30]. In the case of ZnO NRs/PEDOS, the characteristic peak for Zn–O in the ZnO NRs can be found at 635 nm. The absorption peaks of C–O–C bending vibration on PEDOS appear at 1197, 1086 and 1146 cm^−1^.

Additional file 1: Figure S3c shows the XRD of ZnO NRs, PEDOS and ZnO NRs/PEDOS. For ZnO NRs, the diffraction peaks at 2*θ* = 34.5°, 36.3°, 47.5° and 62.9° distribute to (002), (101), (102) and (103) crystal planes of rutile ZnO NRs, respectively. There is a strong and sharp diffraction peak at 34.5°, indicating that the ZnO NRs fabricated by hydrothermal method are a typical fibrous zinc mineral structure with a c-axis selective orientation [31]. It displays that ZnO NRs preferentially grow in the direction perpendicular to the substrate. For PEDOS, it has a broad characteristic peak between 20° and 25°, which is contributed to the amorphous structure caused by the intermolecular π → π* stacking of the PEDOS. After deposition of PEDOS, the diffraction peaks of ZnO and FTO glass prove that the ZnO NRs/PEDOS is successfully prepared. However, it is found that the intensity of (002) diffraction peak of ZnO NRs in ZnO NRs/PEDOS is almost decreased by half for pure ZnO NRs. This can be understood by that after coating PEDOS film on each ZnO nanoarray, the intensity of the diffraction peak in the ZnO NRs/PEDOS hybrid structure decreases.

Additional file 1: Figure S3d is the Raman spectra of ZnO NRs, PEDOS and ZnO NRs/PEDOS. For PEDOS and ZnO NRs/PEDOS, there are characteristic peaks at 1430 cm^−1^, 1480 cm^−1^, 1376 cm^−1^ and 1256 cm^−1^, representing the symmetrical stretching between C_β_ = C_α_ and the stretching between C_α_ = C_α_ and C_β_ = C_β_ of selenium benzene rings, respectively. Additional file 1: Fig. S3d shows the Raman of the samples, and the peak of hexagonal ZnO NRs appears at 434 cm^−1^ [32], which is assigned to the nonpolar optical phonon E2 modes. This is verifying that the wurtzite phase of the ZnO NRs occurred [33].

**Fig. 3:**
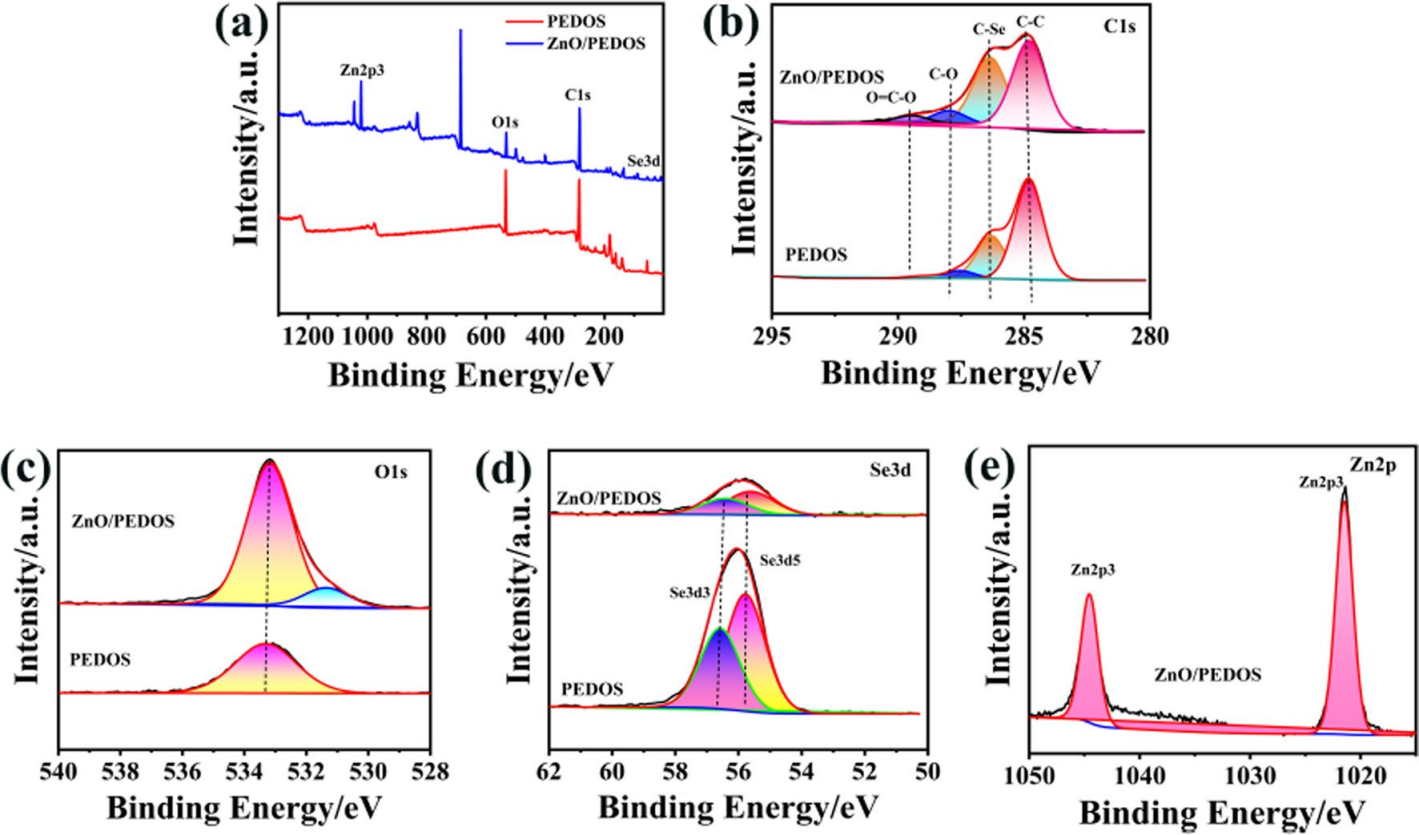
XPS spectra of PEDOS, ZnO NRs/PEDOS composites. **a** Survey, **b** C1*s*, **c** O1*s*, **d** Se3*d*, **e** Zn2*p*

The surface chemical states of PEDOS and ZnO NRs/PEDOS were examined by XPS. Figure 3a shows the survey of PEDOS and ZnO NRs/PEDOS. The Additional file 1: Fig. S4a shows the survey of ZnO NRs, and the peaks at 530.18 eV and 1021.60 eV are assigned to the O1*s* and Zn2*p*3, respectively. There are two peaks at 530.14 eV and 531.48 eV in the Additional file 1: Fig. S4b, and the peak position of the high binding energy corresponds to the internal defect and surface oxygen adsorption signal of zinc oxide, while the peak of the low binding energy corresponds to the O^2−^ signal of zinc oxide [4]. For the energy profile of Zn2*p* exhibit in the Additional file 1: Fig. S4c, the peaks of Zn2*p*3/2 and Zn2*p*1/2 are shown at 1021.78 eV and 1044.77 eV, with a difference of 23 between the two peaks, consistent with that reported in the literature [34].

For PEDOS, the peaks at 283.84 eV, 533.5 eV and 56.97 eV are assigned to the C1*s*, O1*s* and Se3*d*. For ZnO NRs/PEDOS, there are peaks of C1*s*, O1*s*, Se3*d* and Zn2*p*3 at 283.84 eV, 531.73 eV, 56.84 eV and 1021.60 eV, respectively. The binding energy at 685 eV and 498 eV at in the XPS survey spectrum (Fig. 3a) indicates the F 1*s* peak and Sn3*d* that may be appeared because the XPS testing was performed on samples mounted on the FTO glass [35, 36]. Figure 3b shows that there are three peaks in the C1*s* spectra of PEDOS, which is contributed to the signal peaks of C–C, C–Se and C–O bonds. After combination with ZnO NRs, these peaks slightly moved to the higher binding energy direction, implying that the electronic state of polymer is changed by introduction of ZnO NRs. Two peaks at 531.34 eV and 533.18 eV are assigned to C–O and C–O–C bonds of composite in Fig. 3c. Figure 3d is Se3*d* spectrum, and the peaks at 55.77 eV and 56.58 eV are attributed the Se3*d*5 and Se3*d*3 spin splitting doublet of Se atoms. As shown in Fig. 3e, there are two peaks of the Zn2*p* situated at 1021.47 eV (Zn-2*p*_3/2_) and 1044.54 eV (Zn-2*p*_1/2_), which is related to the ZnO [37]. The splitting of the Zn 2*p* peaks (23 eV) indicates that the spin–orbit coupling occurs [38]. Compared with PEDOS, the C1*s*, O1*s* and Se3*d* peaks in the ZnO NRs/PEDOS shift toward low binding energy. These changes in binding energy confirm that there is a chemical interaction between ZnO NRs and PEDOS. This chemical interaction can be understood as the Lewis active centre and hydroxyl group on the ZnO NRs surface, in which the Se atoms with lone pair electrons in PEDOS structure form coordination bonds with Zn atoms by occupying the oxygen vacancy. These interactions effectively improve the photogenerated electron hole transport ability of ZnO NRs in the composite, consequently causing a decrease in the recombination rate of photogenerated electron hole pairs to improve the UV photodetection efficiency of the composites.

There is a built-in electric field at the interface of ZnO NRs/PEDOS composite. The existence of built-in electric field is contributed to the separation of photogenerated carriers, and the existence of built-in electric field is caused by the difference of Fermi energy levels. The Mott–Schottky (M–S) plots were conducted to study the flat band potentials and carrier concentration of ZnO NRs in the composite. The Additional file 1: Fig. S6a–b show M–S plots of ZnO NRs and ZnO NRs/PEODS composite. It can be calculated that the flat band potentials of the ZnO NRs in the ZnO NRs/PEDOS are 0.71 V, it indicates the presence of a strong built-in electric field between ZnO NRs and PEDOS, which is good for the separation of photogenerated electron and hole pairs [39]. According to the literature, the Fermi energy level of ZnO NRs in ZnO NRs/PEDOS heterojunction is calculated to be 3.8 eV. (The calculation process is shown in the Additional file 1.) [40, 41].

**Fig. 4:**
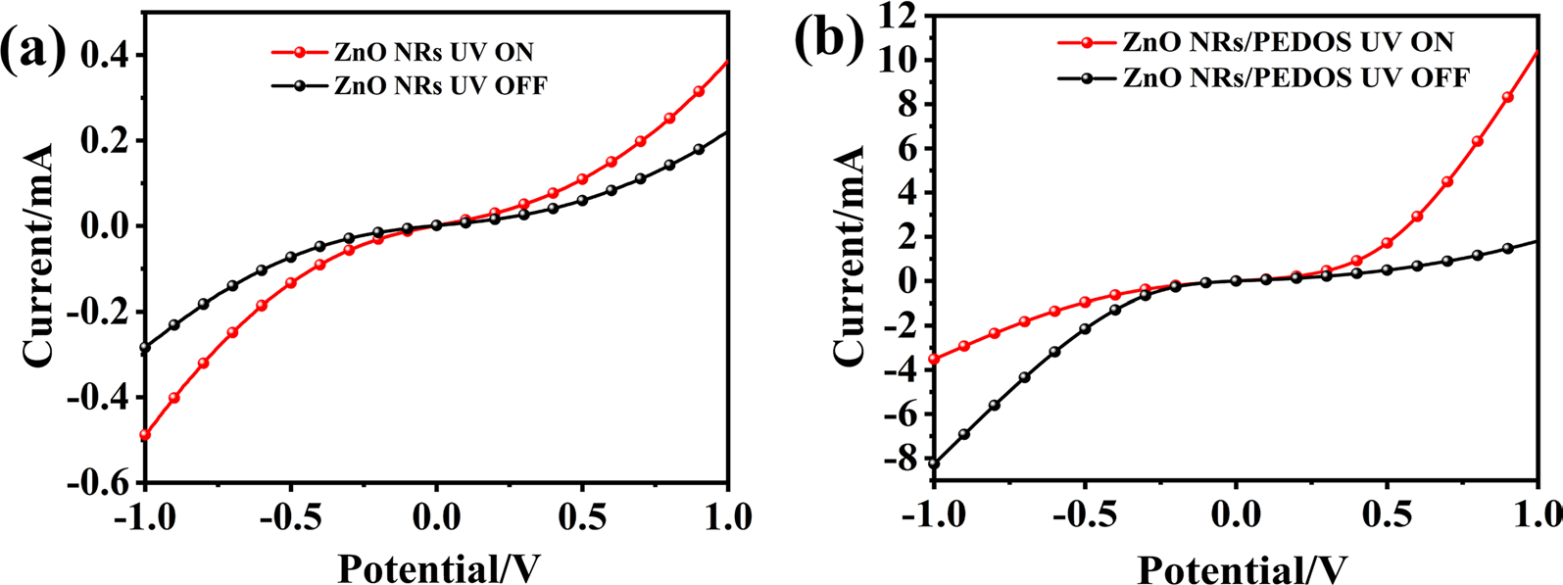
I–V curves of **a** ZnO NRs, **b** ZnO NRs/PEDOS in the dark and under the illuminating at 365 nm UV light

Figure 4(a), (b) shows the I–V characteristic curves of ZnO NRs and ZnO NRs/PEDOS UV PDs under dark condition and 365 nm UV light at the bias voltage of − 1 to + 1 V. They indicate that these devices exhibit good rectification behaviors under 1 V bias voltages [42]. The photocurrent of ZnO NRs/PEDOS UV PD has a significant enhancement comparing with that of ZnO NRs UV PD. The reason this phenomenon in photocurrent of composite can be explained by the role of PEDOS as a hole transport material.

When PEDOS contacts with ZnO NRs, forming a depletion layer near the material contact surface, the direction of the built-in electric field is determined from ZnO NRs to PEDOS p-n interface. Under UV light, the photogenerated electrons and holes generated in the depletion layer are separated in the internal electric field, which can achieve the purpose of enhancing the photocurrent. Another reason is that the p-n heterojunction effect between PEDOS and ZnO results in the different rectifying performances of these UV PDs under dark and ultraviolet light conditions.

An important parameter of photodetector is responsivity (*R*), which indicates the ability of photodetector to convert light into photocurrent. To evaluate the behavior of UV PD, the physical parameters such as detection rate and external quantum efficiency are discussed. Further mathematical characterization is carried out according to formulas (1), (2) and (3) [2, 43, 44]. The detailed values can be seen in Table 1

1 $$R = \frac{{I_{{{\text{ph}}}} - I_{{{\text{dark}}}} }}{P \times S}$$

2 $$D = \frac{R}{{\sqrt {2eI_{{{\text{dark}}}} {/}S} }}$$

3 $${\text{EQE}} = \frac{R \times hc}{{e\lambda }}$$

Among them, *S* is the effective light area of the device, *P* is the ultraviolet light intensity, *I*_ph_ and *I*_dark_ are the current measured by the device under dark and ultraviolet light, h is constant of Planks, *e* is elementary charge and *c* is the light speed.

**Table 1:** Performances of ZnO NRs and ZnO NRs/PEDOS UV PDs under 1 V bias

Sample	*I*_ph_ (mA)	*I*_dark_ (mA)	*R* (A/W)	D* (Jones)	EQE (%)
ZnO NRs	0.293	0.108	1.76	1.64 × 10^11^	600
ZnO NRs/PEDOS	11.86	0.50	108.2	4.68 × 10^12^	36,800

**Fig. 5:**
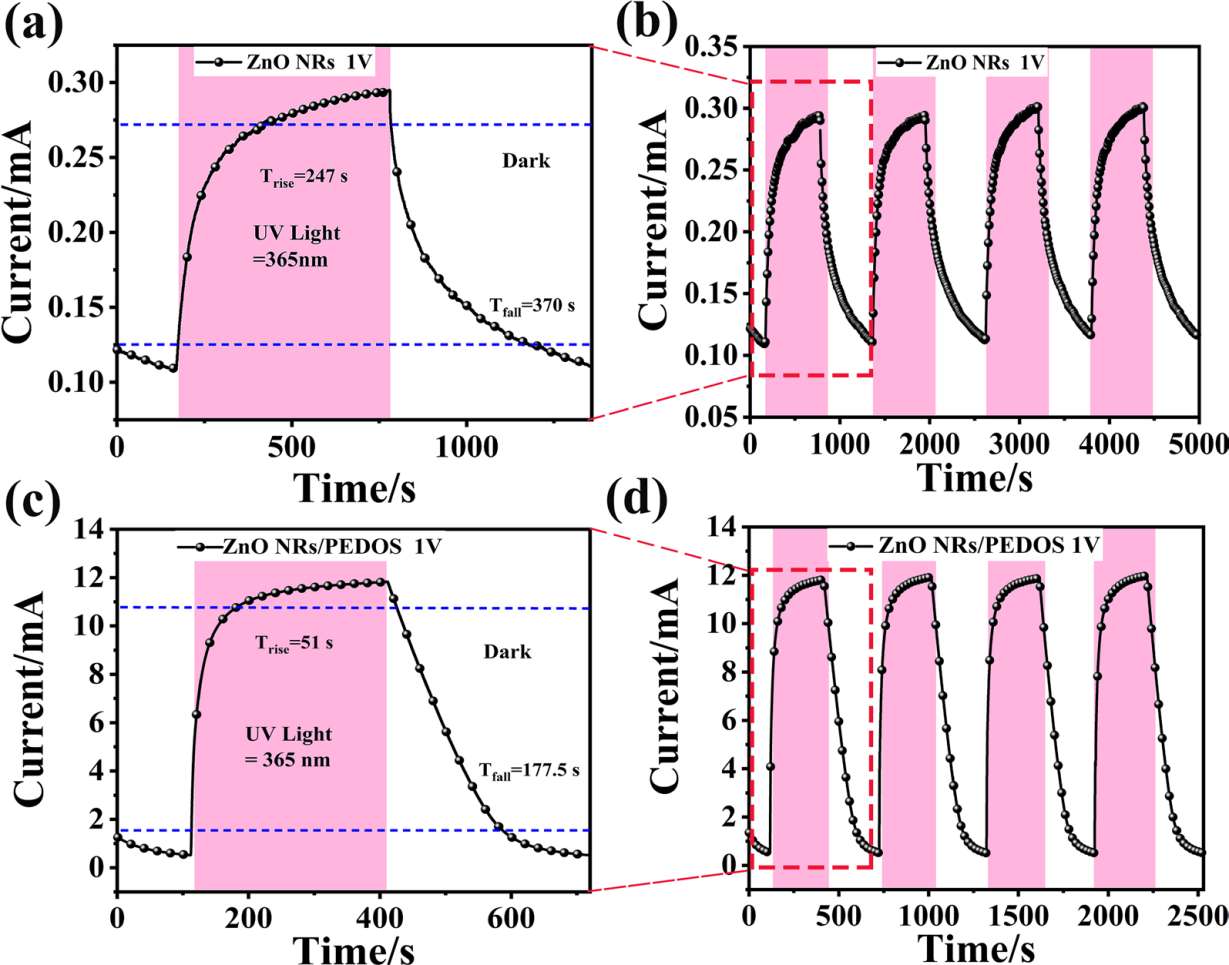
**a**, **b** single-cycle I–T curve and multicycle I–T curve of ZnO NRs UV PD. **c**, **d** single-cycle I–T curve and multicycle I–T curve of ZnO NRs/PEDOS UV PD

Response time is affected by photoelectron–hole pair recombination probability and transmission rate, which is another important parameter to evaluate the performance of photodetector [44]. In order to investigate the effect of PEDOS on the performance of the prepared device, the transient light corresponding (I–T) curve of the ZnO NRs/PEDOS heterojunction ultraviolet photodetector was studied at the light power of 0.3 mW/cm^2^ and 1 V bias voltage. The response speed of the UV PD can be expressed by *τ*_rise_ and *τ*_fall_. The *τ*_rise_ represents the time of the photoelectric current rise from 10 to 90% of the stable current, while the *τ*_fall_ represents the time decreasing from 90 to 10% of the maximum photocurrent [45]. As can be seen from Fig. 5a, c, the dark current and photocurrent of the ZnO NRs UV PD are 0.108 mA and 0.293 mA, respectively. Compared to ZnO NRs UV PD, the dark current and photocurrent increase to varying degrees, and current values are 0.5 mA and 11.86 mA, respectively.

As seen from Fig. 5b, d, when the UV light source is turned on, the photocurrent of the p-n hybrid structure heterojunction ultraviolet detector increases rapidly; when the ultraviolet light source is turned off, the photocurrent decreases rapidly. In addition, the ZnO NRs/PEDOS p-n heterojunction ultraviolet detector has maintained good stability after four I–T cycles.

The *τ*_rise_ and *τ*_fall_ values of the UV PD made of pure ZnO NRs are 247 s and 370 s, respectively (Fig. 5a). Compared with the ZnO NRs UV PD, the response time of the ZnO NRs/PEDOS is shortened a lot, and the response time is 51 s (*τ*_rise_) and 177.5 s (*τ*_fall_), respectively (Fig. 5c). In addition, the ZnO NRs/PEDOS showed significant higher photodetector performance. This is not only due to that the PEDOS has excellent carrier transport and reduces the probability of electron–hole recombination [24], but also due to the core–shell structure of ZnO NRs/PEDOS, which is the ZnO NRs coated with PEDOS films to effectively isolate the air on the surface of ZnO NRs, reducing the adsorption and absorption of oxygen molecules, thereby reducing recovery time.

**Fig. 6:**
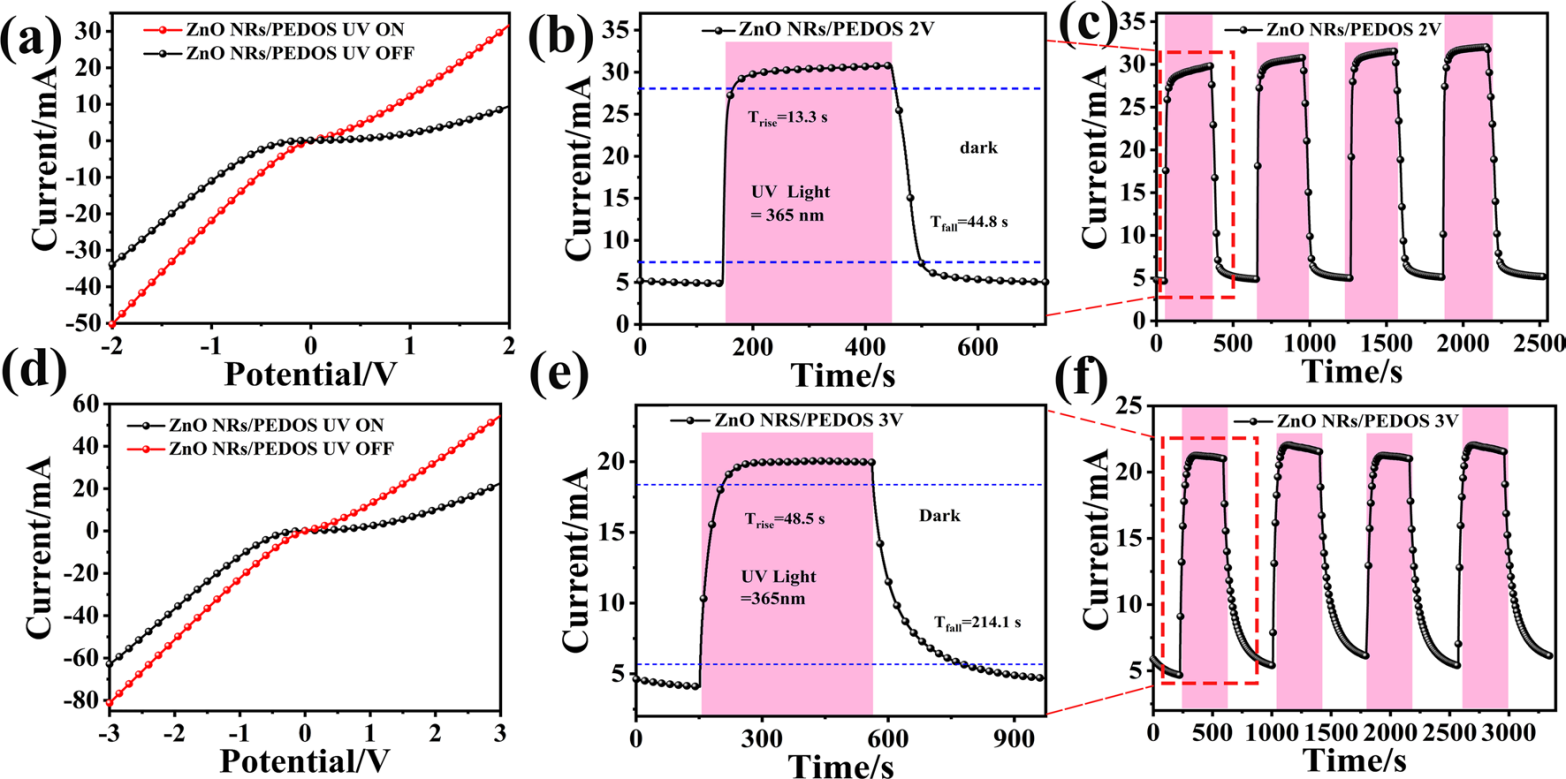
I–V curves (**a**) current–time (**b**) and circulating current–time (**c**) diagram of ZnO-NRs/PEDOS UV PD under bias 2 V and I–V curves (**d**) current–time (**e**) and circulating current–time (**f**) diagram of ZnO-NRs/PEDOS UV PD under different bias 3 V

For further analysis, the performance curve of the ZnO NRs/PEDOS UV PD was tested at bias voltage with different values (2–3 V). Figure 6 shows that the device maintains good p-n heterojunctions between p-type PEDOS and n-type ZnO NRs under different voltages, which illustrates enhancement in the photocurrent under different bias voltages. After four cycles, the response remains unchanged. As the voltage increases from 2 to 3 V, the *τ*_rise_ decreases gradually.

Figure 6 depicts the change of photodetector performance under bias voltage, and the specific values are shown in Table 2. As the bias voltage increases, the p-n heterojunction barrier of ZnO-NRs/PEDOS gradually decreases, which is resulted from the acceleration of the diffusion of carriers to form photocurrent, and the responsivity also increases, as well as the detectivity, external quantum efficiency and responsivity. The best performance is obtained under 2 V bias voltage, and the responsivity is 247.7 A/W with the quantum efficiency of 84,000%.

**Table 2:** Performance comparison of different ZnO-based UV PDs

materials	Bias (V)	Wavelength (nm)	Light intensity (mW/cm^2^)	*R* (A·W^−1^)	D* (Jones)	EQE (%)	Refs
n-ZnO/p-GaN	0	274	–	1 × 10^−6^	–	–	[47]
ZnO/polyaniline/ZnGa_2_O_4_	0	365	–	–	–		[48]
Mg doped p-GaN/PVA-ZnO	0	350	–	2.25 × 10^−5^	–		[49]
ZnO/PEDOT:PSS	5	360	100	0.403	3.22 × 10^8^	–	[26]
PANI/ZnO MW	− 3	350		0.037	–		[17]
Ppy-PEDOT:PSS/GaN NRs	0	382	6.56	110	3.08 × 10^13^	4.0 × 10^5^	[50]
ZnO-polyaniline	3	365	–	–	–	–	[15]
ZnO/GQD/PEDOT:PSS	− 1	340	–	36	–	–	[51]
ZnO nanorods/polyaniline	5	400	0.932	0.039	–	12.28	[52]
ZnO-NRs/GaN-NTs	6	325	3.2	1204	1.84 × 10^14^	2.7 × 10^6^	[44]
ZnO NRs/PEDOS	2	365	0.3	247.7	3.50 × 10^12^	84,000	This work

However, when bias voltage reaches 3 V, it can be found that the photocurrent and responsivity decreases, because the depletion layer disappears which results the performance to decrease [46]. In addition, the trap of ZnO is gradually filled with the photogenerated carriers of the PEDOS, resulting in a reduce in the average carrier life time. Thus, the response rate of the UV PD decreases with increasing of applied bias voltage. The performance comparison with the previously reported UV detectors is listed in Table 2.

We compared the ZnO NRs/PEDOS heterojunction device with the reported relevant photodetectors, as shown in Table 2. Heterojunction photodetectors composed of n-ZnO and p-polymers have been less reported, and the photodetectors that have been reported also have problems such as low response and detection rate in the higher light intensity. Therefore, the ZnO NRs/PEDOS heterojunction ultraviolet detector exhibits high response and detection rate at lower light intensities.

Stability of the device by measuring responsivity under the light intensity 0.32 mW/cm^2^ at 2 V for 15 days measured the performance daily and observed the changes in the responsiveness. The results showed (Additional file 1: Fig. S7) that the prepared ZnO NRs/PEDOS UV PD showed better stability.

**Fig. 7:**
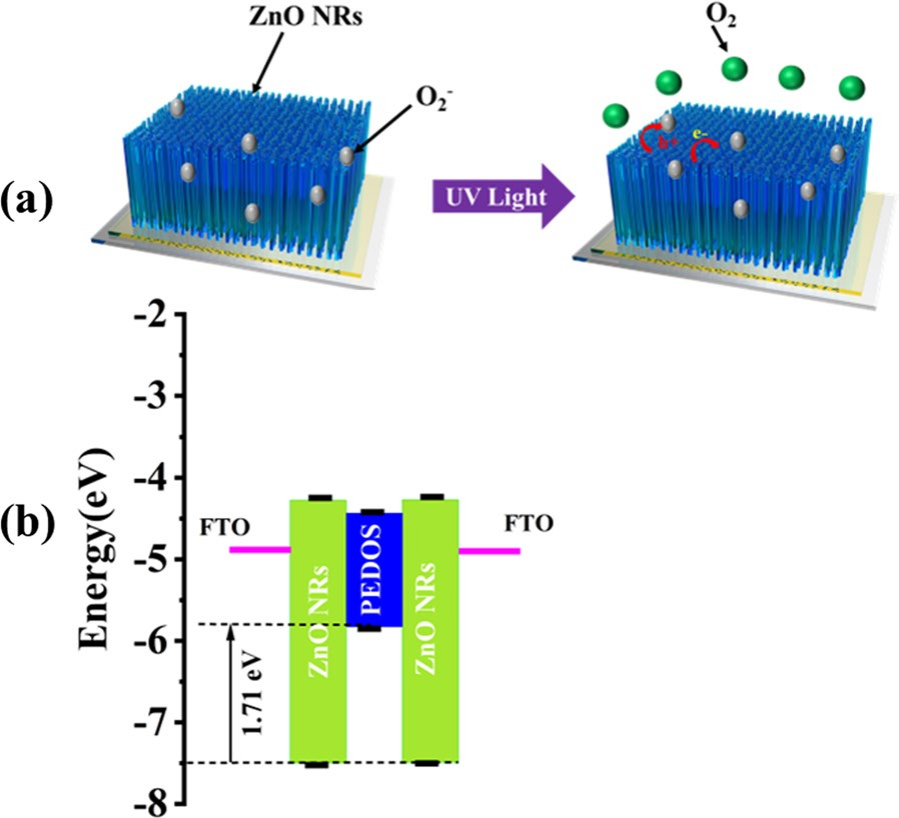
**a** The oxygen adsorption/desorption process of ZnO NRs. **b** The scheme of energy band of ZnO NRs/PEDOS p-n UV PD

Based on the report [53], the E_HOMO_ and E_LUMO_ of PEDOS are − 5.78 eV and − 4.38 eV, respectively. For ZnO, the valence band (VB) and conduction band (CB) values are obtained from published papers [54]. Figure 7a shows that the photoconductor of pure FTO/ZnO NRs structure (without PEDOS) works by slow oxygen adsorption/desorption modulated at the UV irradiation. In the dark, oxygen molecules in the air adsorb on the surface as O^2−^ ions, reducing the conductivity of ZnO NRs [55–57]. Under UV light, ZnO NRs produce a lot of electron-pore pairs. Meanwhile, the O^2−^ ions of ZnO NRs combine with the charged holes to arose a O_2_ gas, forming free electrons as the main charge carrier under the external bias [35, 36].

Figure 7b and Additional file 1: Fig. S8 shows the band diagram and the HOMO/LUMO energy level of the UV PD of ZnO NRs/PEDOS [37, 38, 58]. Since these materials have different band gaps and work functions, this leads to the bending of the energy band [59]. The potential barrier of VB edge between ZnO NRs and PEDOS is 1.7 eV, and the hole injection barrier is low. This shows that photogenerated holes can be easily injected from ZnO NRs VB into PEDOS holes [22], [60]. This can be a reason why the ZnO NRs/PEDOS heterojunction shows good responsivity behaviors.

ZnO NRs/PEDOS forms a p-n heterojunction, in which PEDOS as a p-type material forms a hole depletion region and ZnO NRs as an n-type material includes an electron depletion region. A potential difference is established at the ZnO NRs/PEDOS interfacial heterojunction due to the different energy band gaps of the two materials. The working mechanism is elucidated by carefully study the energy band diagram of the ZnO NRs/PEDOS interface, that is, the underlying physical properties. When a p-n heterojunction was established from ZnO NRs/PEDOS by UV irradiation, the carriers on the ZnO NRs surface are excited and electrons–hole pair are formed in the depletion region. Owing to the built-in electric field is cancelled under the bias voltage, therefore, photogenerated electrons are transferred to the ZnO NRs layer and holes are transferred to the PEDOS layer to enhance the photocurrent. There are oxygen vacancies on the ZnO NRs surface [61], and the defect vacancies are decreased by the hybridization of PEDOS with ZnO NRs, and the number of free electrons on the ZnO NRs is increased. Under the external bias voltage, the p-n heterojunction barrier is reduced, which is conducive to the diffusion of carriers to form a larger current for enhancing the optical response. Therefore, PEDOS-based UV PD has good performance (high photocurrent and excellent response speed).

## Conclusion

In this paper, ZnO NRs/PEDOS core–shell composite was fabricated by electrochemical deposition of PEDOS on the surface of the ZnO NRs to fabricate the p-n heterojunction UV PD. The core–shell structure of ZnO NRs/PEDOS composite enabled the formation of an effective heterojunction between ZnO NRs and PEDOS, which was benefit for the generation, transmission and separation of photogenic electron pairs to improve the photoelectric properties of the composites. It was found that the PEDOS played as hole transport material transfer photoelectrons, and it inhibited the recombination of photoelectron–hole pairs, which was good for improve the performance of UV PD. Moreover, the energy level of PEDOS matching with ZnO NRs was conducive to the formation of strong ionized electric fields. The coupling between p-type low band gap PEDOS and n-type ZnO NRs effectively hindered the recombination of photogenerated electron–hole pairs, thus enhancing the performance of the detector. As a result, the *R*, D* and external quantum efficiency (EQE) of the UV PD reached to 247.7 A/W, 3.41 × 10^12^ Jones and 84,000% at 2 V bias, respectively. It is indicated that the introduction of PEDOS effectively improved the performance of the UV PD.

## Supplementary Information

**Additional file 1**. The synthetic route of EDOS monomer and ^1^H-NMR spectra of 3,4-dimethoxyselenophene and EDOS in CDCl_3_; CV curve of PEODS grown on ZnO NRs by electrochemical deposition; UV-vis, FTIR, XRD spectra and Raman spectra of ZnO NRs, PEDOS, ZnO NRs/PEDOS; XPS spectra of ZnO NRs; Absorption spectrum for ZnO NRs and PEDOS; M–S curves of ZnO NRs and ZnO NRs/PEDOS; Stability of the device; Working mechanism of the device.

## Data Availability

All data generated or analyzed during this study are included in this published article [and its supplementary information files].
