# Supplementary material for: Ultraviolet Photodetector Based on Poly(3,4-Ethylenedioxyselenophene)/ZnO Core–Shell Nanorods p-n Heterojunction
Source: Nanoscale Res Lett. 2022 Jul 25;17:67. doi: 10.1186/s11671-022-03705-4 (PMC9314489; doi:10.1186/s11671-022-03705-4)
Supplement: Supplementary file 1 — Additional file 1. The synthetic route of EDOS monomer and 1H-NMR spectra of 3,4-dimethoxyselenophene and EDOS in CDCl3; CV curve of PEODS grown on ZnO NRs by electrochemical deposition; UV-vis, FTIR, XRD spectra and Raman spectra of ZnO NRs, PEDOS, ZnO NRs/PEDOS; XPS spectra of ZnO NRs; Absorption spectrum for ZnO NRs and PEDOS; M–S curves of ZnO NRs and ZnO NRs/PEDOS; Stability of the device; Working mechanism of the device. [file 11671_2022_3705_MOESM1_ESM.docx]

Additional file 1

**Ultraviolet Photodetector based on poly (3,4-ethylenedioxyselenophene)/ZnO core-shell Nanorods p-n Heterojunctions**

Aygul Kadir^a,b^, Ruxangul Jamal^b^, Tursun Abdiryim^a*^,Xiong Liu^a^, Hujun Zhang^a^, Dongna Zou^a^ ,Nawrzhan Serkjan^a^_，_Ya jun Liu^a^

^a^ State Key Laboratory of Chemistry and Utilization of Carbon Based Energy Resources; College of Chemistry, Xinjiang University, Urumqi, 830017, Xinjiang, PR China.

^b^ State Key Laboratory of Chemistry and Utilization of Carbon Based Energy Resources, Key Laboratory of Petroleum and Gas Fine Chemicals, Ministry of Education, College of Chemical Engineering, Xinjiang University, Urumqi, 830017, Xinjiang, PR China.

* Corresponding author: Tel: +86 09918582809; fax: +86 09918582809

E-mail: [tursunabdir@sina.com.cn](mailto:tursunabdir@sina.com.cn)

**Preparation of the ZnO NRs**

According to previously reported literature^1^, ZnO NRs were grown on FTO conducting glass by hydrothermal method. The fluorine-doped tin oxide (FTO) Glass substrates were initially ultrasonic cleaned with acetone, ethanol and deionized water, successively, and then blown dry with dry air. ZnO seed layer was firstly deposited on the fluorine-doped tin oxide (FTO) glass by spin-coating process. Preparation of zinc oxide solvent: dissolve 0.5488 g zinc dihydroacetate in 50 ml ethanol. At the 60℃ the amount of eththanolamine (about 0.15 ml) with zinc is added to the above solution and stirred for 2 h to stabilize the transparent solvent. An aqueous solution for ZnO growth was prepared with 25 mM hexamethylenetetramine and 25 mM zinc nitrate. A piece of FTO substrate with the ZnO seed layer was placed at an angle against the wall of the autoclave with the conducting side facing down. The autoclave was sealed and placed in an oven at 95 C for 4 h before the sample was rinsed with deionized water.

**Synthesis of EDOS**

2,3-dimethoxy-1,3-butadiene was synthesized according to a previously reported method^2^.

EDOS was synthesized according to a previously reported method^3^. First prepared SeCl_2_, SO_2_Cl_2_ and selenium powder were as reactants, and n-hexane as solvent. A solution of freshly-prepared SeCl_2_ in hexanes was added to a well-stirred mixture of 2,3-dimethoxy-1,3-butadiene (24.0 g, 210 mmol) and CH_3_COONa (8.2 g, 100 mmol) (as the buffering agent) in hexanes (250 mL) (as the solvent) at –78 °C under an inert atmosphere. The resulting yellowish solution was further stirred for 1 h at -78 °C and the reaction mixture was warmed to room temperature stirred for another 4 h. The reaction mixture was filtered and washed with n-hexane. The residue was concentrated to provide a brown oil that was purified by recrystallization in hexanes at low temperature to furnish a white crystalline solid. 1H NMR (400 MHz, CDCl_3_): δ (ppm) = 6.55 (s, 2H), 3.85 (s, 6H).

A solution of 3,4-dimethoxyselenophene (1 g, 5.24 mmol), with 6 equivalents of ethylene glycol (2 g, 32.41 mmol) and a *p*-toluene sulfonic acid (160 mg) (as catalytic agent) in dry toluene (150 mL) was stirred for 12 h at 50 – 55 °C. Toluene was removed under reduced pressure, and the residue was diluted with water (100 mL). The mixture was extracted with dichloromethane (3 x 50 mL). The combined organic layers were washed with dilute NaCl solution and brine and then Spinning solvent to concentrated. Purification of the crude residue by silica gel chromatography gave 3,4-ethylenedioxyselenophene as a colorless liquid. 1H NMR (400 MHz, CDCl3): δ (ppm)= 6.79 (s, 2H), 4.17 (s, 4H).


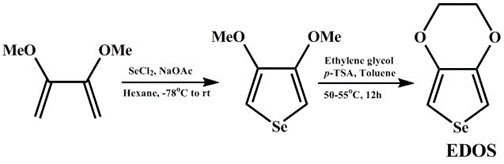


**Additional file 1: Scheme 1.** The synthetic route of EDOS monomer


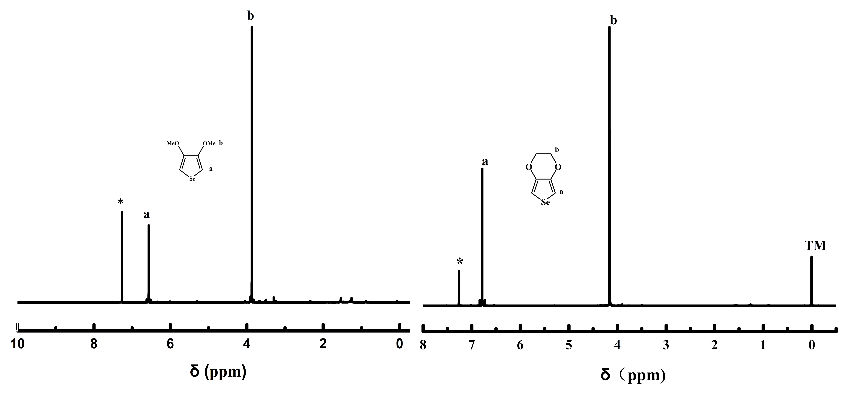


**Additional file 1: Fig. S1.** 1H-NMR spectra of 3,4-dimethoxyselenophene and EDOS in CDCl_3_


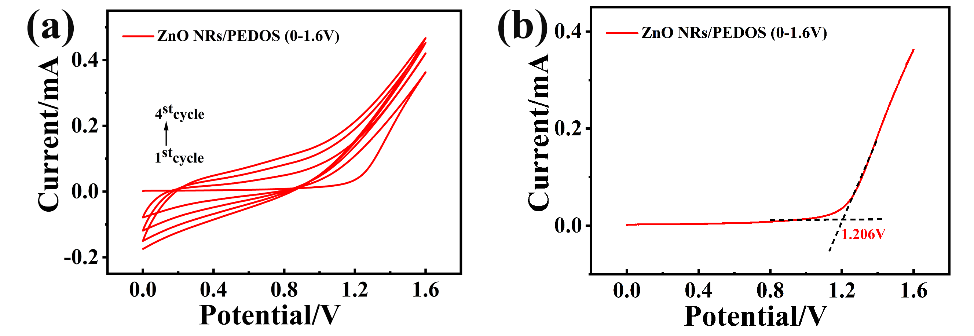


**Additional file 1: Fig. S2.** (a, b) CV curve of PEODS grown on ZnO NRs by electrochemical deposition

**Assembly of UV-light detectors**

The photodetector was assembled to a sandwich structure, the process as follows: ZnO NRs was used as the counter electrode, and the working electrode is ZnO NRs grown with a layer of polymer PEDOS. The two electrodes were tightened with clamps, and then the performance of the UV detector was tested. The voltage-current and current-time curves of the detector were tested at a wavelength of λ=365 nm and a light intensity of 0.32 mW/cm^2^ with an effective light area of 0.3 cm^2^.


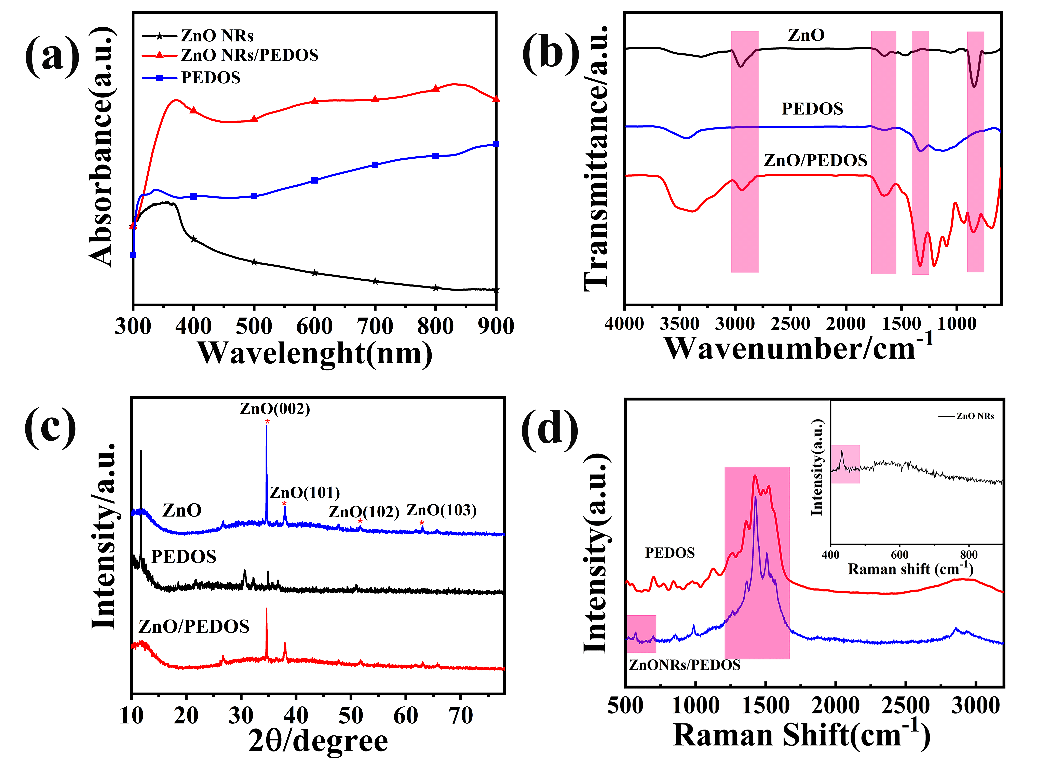


**Additional file 1: Fig S3** a UV-vis, b FT-IR, c XRD spectra d Raman spectra of ZnO NRs, PEDOS, ZnO NRs/PEDOS composite


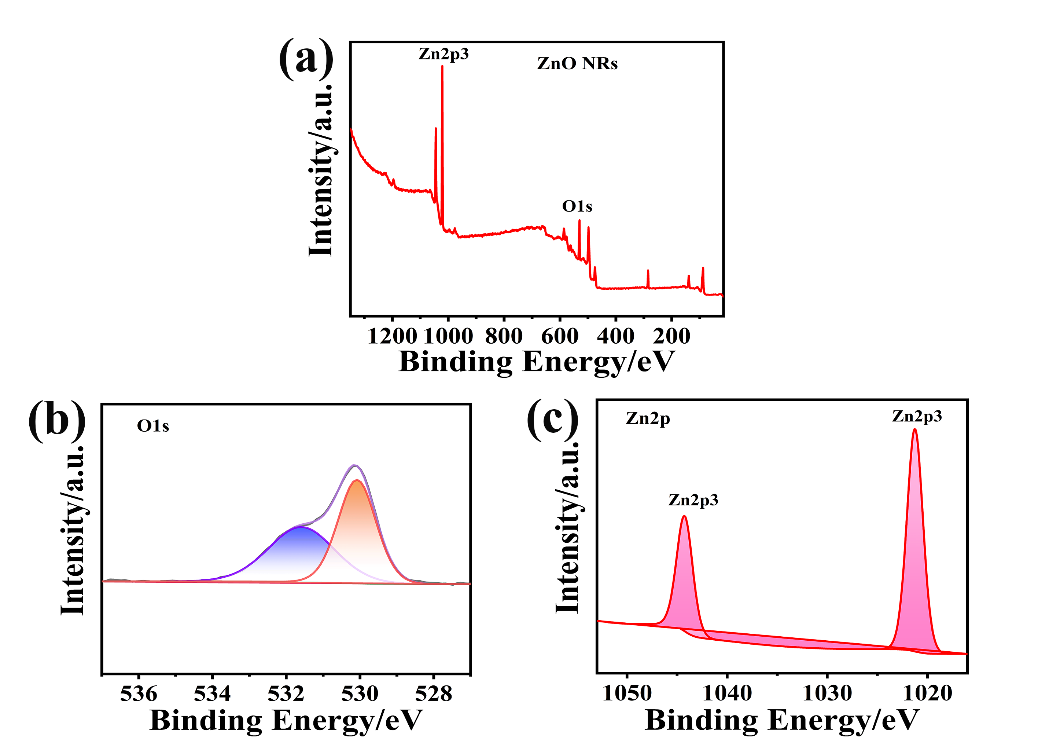


**Additional file 1: Fig. S4.** XPS spectra of ZnO NRs **(a)** survey **(b)** O1s **(c)** Zn2p.

Spectra of ZnONRs and ZnONRs / PEDOS deposited on FTO were used to assess the absorption characteristic properties (Figure S4a, b) and the band gap values of the layers (the inset of Figure S4(a), (b) shows Tauc diagram). The optical bandgap values are found to be 3.15 and 1.45 eV for ZnONRs and PEDOS, respectively, as seen from the Tauc plot.


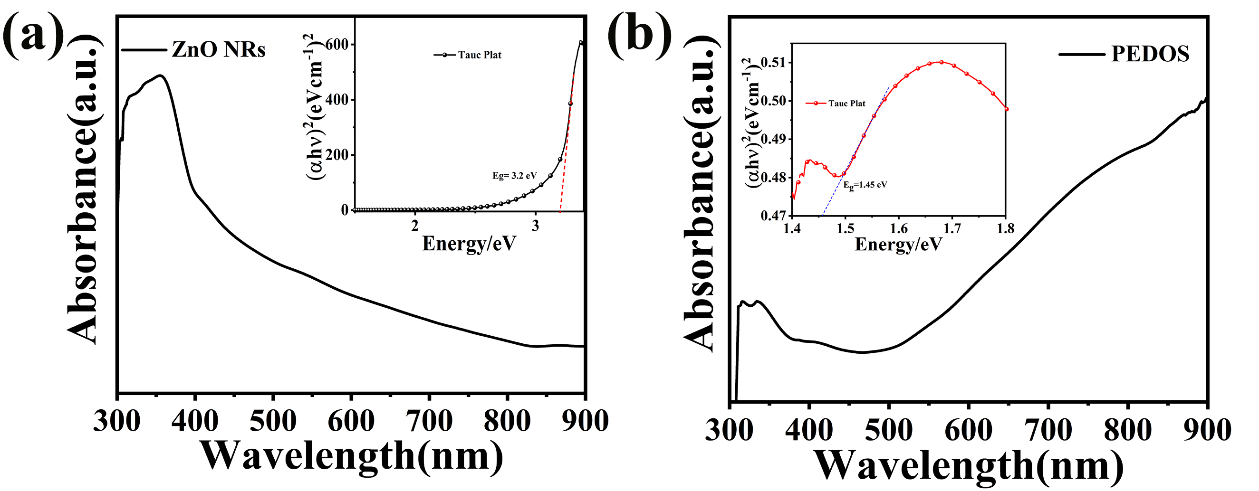


**Additional file 1: Fig. S5.** Absorption spectra (inset shows Tauc plots) for ZnO NRs and PEDOS


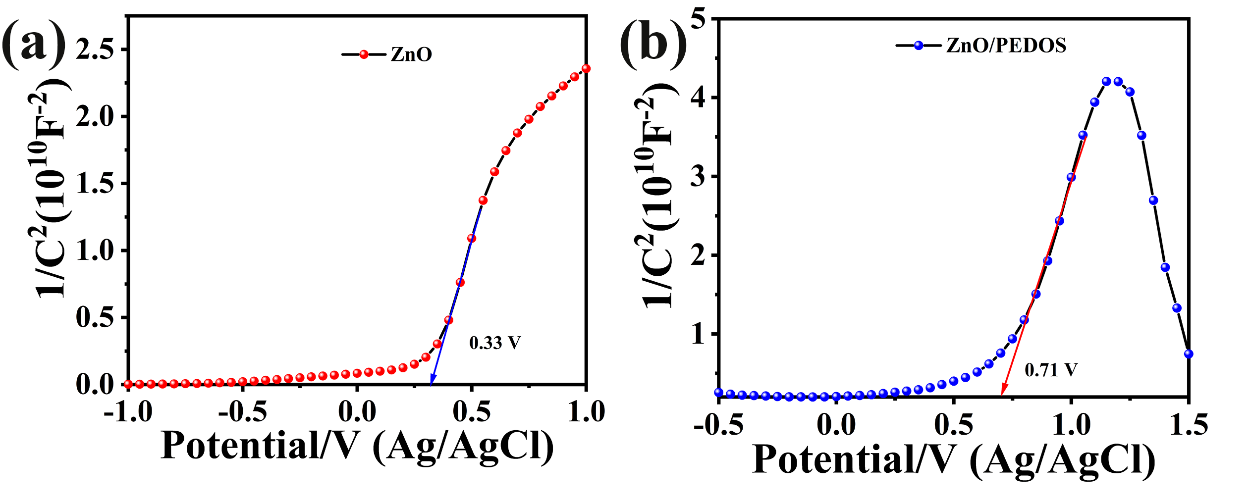


**Additional file 1: Fig. S6.** M–S curves for (a)ZnO NRs, (b) ZnO NRs/PEDOS

Fig. S5 shows the Mott–Schottky (M–S) curves of ZnO NRs, ZnO NR_S_/PEDOS. It can be seen from the figure that ZnO NR_S_ are a n-type semiconductor, while the polymer is a p-type semiconductor^4^. The flat band potentials of ZnO NRs/PEDOS heterojunctions were 0.71 V (the intercept of the fitting line in the figure on the x-axis). The flat band potential of ZnO NRs/PEDOS heterojunction is the large, indicating that there is a strong built-in electric field in the heterojunction, which can effectively separate photogenerated electrons and hole pairs, resulting in the improvement of photodetector performance^5^.

The calculation equations for carrier concentration (N_D_) of ZnO in the ZnONRs/PEDOS heterojunctions can be estimated from formula (1)^5^:

$$N_{D}=\pm\left( \frac{2}{q\varepsilon_{r}\varepsilon_{0}A^{2}} \right) \left[ \frac{d\left( 1/C^{2} \right)}{dV} \right]^{-1} (1)$$

where q is electronic charge (1.602 × 10^−19^ C), ε_r_ is the relative dielectric constant of sample (ε_r_‑ZnO = 8.86), ε_0_ is vacuum dielectric constant (ε_0_ = 8.854 × 10^−12^ F/m), A is the effective working area (A = 0.3 cm^2^), C is capacitance, and V is the applied potential at the electrode, V_FB_ is flat band potential of ZnO NRs in the ZnONRs/PEDOS heterojunctions.

The values of potential difference between the conduction band edge and the Fermi level (E_C_ – E_F_) of ZnO NRs in the ZnO NRs/PEDOS heterojunctions is calculated by formula (2).

$$\frac{E_{C}-E_{F}}{q}=\frac{KT}{q}\ln\left( \frac{N_{C}}{N_{D}} \right) (2)$$

Here, Nc refer to the effective density of the conduction band^6^. The calculations of barrier height ($\phi_{B,eff}$) in the ZnO NRs/PEDOS heterojunctions can be estimated from the semi-logarithmic I-V curves and formula (3)^7^:

$$\phi_{B,eff}= \frac{KT}{q}\ln\left( \frac{AA^{*}T^{2}}{I_{o}} \right) (3)$$

where A is the junction area (0.3 cm^2^), A* is the effective Richardson constant (≈1200 Acm^−2^ K^−2^ for ZnO), I_o_ is the reverse saturation current, K is the Boltzmann constant, and T is the absolute temperature.

The Fermi levels of ZnO NRs/PEDOS is -3.8 eV.


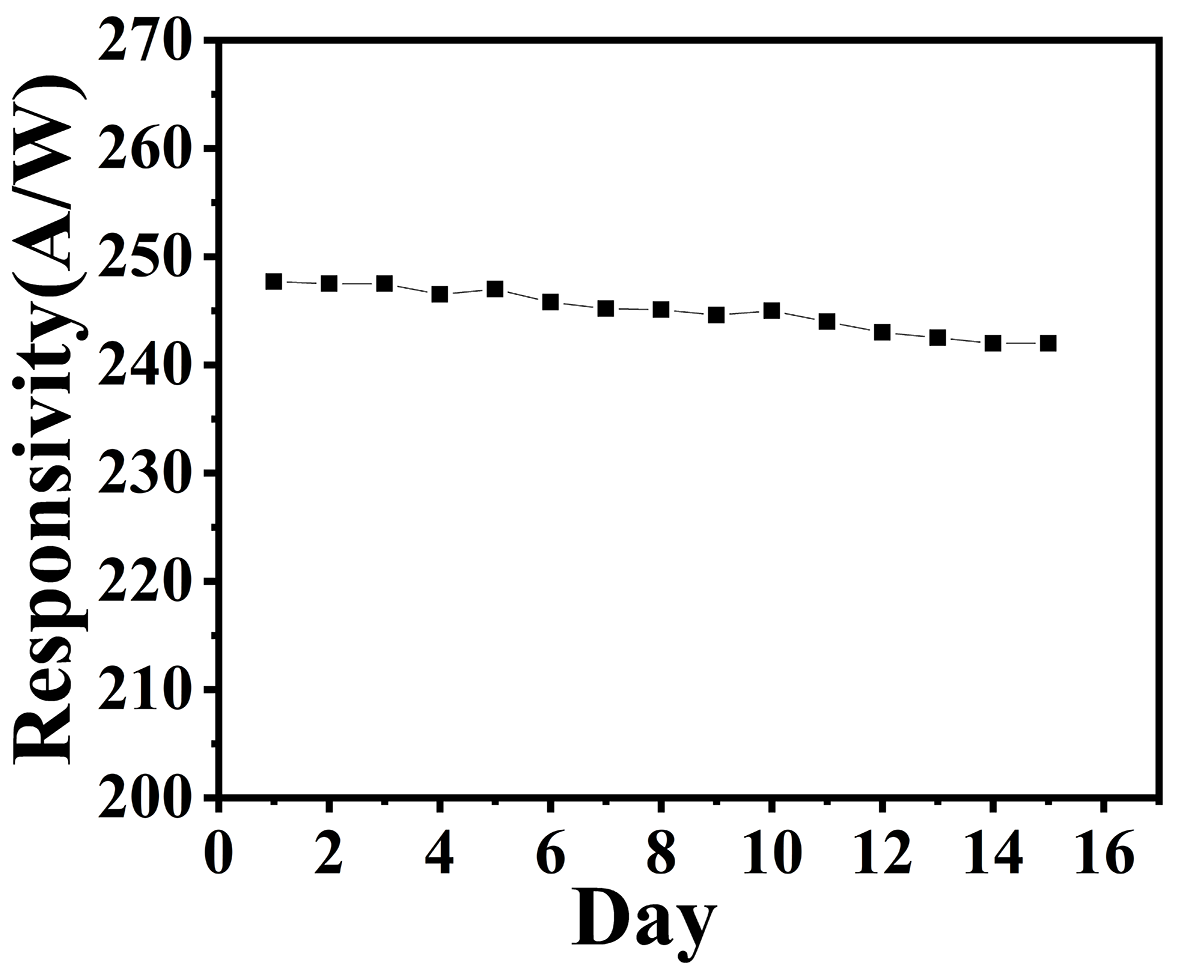


**Additional file 1: Fig. S7**. Stability of the device


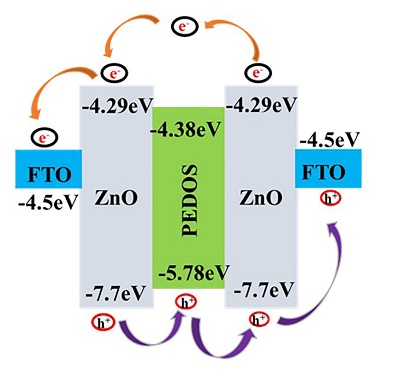


**Additional file 1: Fig. S8**. Working mechanism of the device

**References**

1 J. Ding, H. Chen, D. Feng and H. Fu J. Journal of Alloys and Compounds, 2017, **714**, 198–203.

2 J. L. Pozzo, G. M. Clavier, M. Colomes and H. Bouas-Laurent, *Tetrahedron*, 1997, **53**, 6377–6390.

3 A. Patra, Y. H. Wijsboom, S. S. Zade, M. Li, Y. Sheynin, G. Leitus, M. Bendikov and V. Reho, 2008, 6734–6736.

4 M. H. Tran, T. Park and J. Hur, *Appl. Surf. Sci.*, 2021, **539**, 148222.

5 C. Wei, J. Xu, S. Shi, Y. Bu, R. Cao, J. Chen, J. Xiang, X. Zhang and L. Li, *J. Colloid Interface Sci.*, 2020, **577**, 279–289.

6 P. Lin, X. Yan, Z. Zhang, Y. Shen, Y. Zhao, Z. Bai and Y. Zhang, *ACS Appl. Mater. Interfaces*, 2013, **5**, 3671–3676.

7 B. Ezhilmaran, M. Dhanasekar and S. V. Bhat, *Nanoscale Adv.*, 2021, **3**, 1047–1056.
